# Liquid Biopsy in Rare Cancers: Lessons from Hemangiopericytoma

**DOI:** 10.1155/2018/9718585

**Published:** 2018-03-07

**Authors:** Chiara Nicolazzo, Luciano Colangelo, Alessandro Corsi, Guido Carpino, Angela Gradilone, Chiara Sonato, Cristina Raimondi, Eugenio Gaudio, Paola Gazzaniga, Walter Gianni

**Affiliations:** ^1^Department of Molecular Medicine, Circulating Tumor Cells Unit, Sapienza University of Rome, Viale Regina Elena 324, 00161 Rome, Italy; ^2^Policlinico Umerto I, II Division of Internal Medicine and Geriatrics, Sapienza University of Rome, Viale del Policlinico 155, 00161 Rome, Italy; ^3^Department of Radiological, Oncological and Anatomopathological Sciences, Sapienza University of Rome, Viale Regina Elena 324, 00161 Rome, Italy; ^4^Department of Movement, Human and Health Sciences, Division of Health Sciences, Foro Italico University of Rome, Piazza Lauro De Bosis 6, 00135 Rome, Italy; ^5^Department of Anatomical, Histological, Forensic Medicine and Orthopedics Sciences, Sapienza University of Rome, Via Alfonso Borelli 50, 00161 Rome, Italy

## Abstract

Hemangiopericytoma (HPT) is a rare mesenchymal tumor of fibroblastic type and for its rarity is poorly studied. The most common sites of metastatic disease in patients with intracranial HPT are the bone, liver, and lung, suggestive for an hematogenous dissemination; for this reason, we investigated, for the first time, the presence of circulating tumor cells (CTCs) in hemangiopericytoma patient by CellSearch® and SceenCell® devices. Peripheral blood samples were drawn and processed by CellSearch, an EpCAM-dependent device, and ScreenCell®, a device size based. We found nontypical CTCs by CellSearch system and the immunofluorescence analysis performed on CTCs isolate by ScreenCell demonstrated the presence of single CTCs and CTC clusters. The molecular characterization of single CTCs and CTC clusters, using antibodies directed against EpCAM, CD34, cytokeratins (8, 18, and 19), and CD45, showed a great heterogeneity in CTC clusters. We believe that the present study may open a new scenario in the rare tumors: the introduction of the liquid biopsy and the molecular characterization of circulating tumor cells could lead to personalized targeted treatments and also for rare tumors.

## 1. Introduction

Hemangiopericytoma (HPT) is a rare mesenchymal tumor of fibroblastic type [1]. Although originally presumed to originate from vascular pericytes [2], the precise histogenesis has not been definitely established. From a mere clinical point of view, HPT represents an unpredictable disease, since distant relapses are usually delayed and may occur even decades after diagnosis of the primary tumors [3], suggesting that a long-term follow-up is strongly needed [4]. Treatments are limited so far also due to inadequate funding for preclinical and clinical research programmes, and new strategies are mandatory in order to allow the earlier detection of tumor recurrence and metastasis, thus improving the diagnosis, treatment, and surveillance of patients. The most common sites of metastatic disease in patients with intracranial HPT are the bone, liver, and lung, suggestive for an hematogenous dissemination [5]. Circulating tumor cells (CTCs) represent a unique source of information about hematogenous spread of tumors and are recognised as a useful tool in the identification of new clinically relevant biomarkers [6]. Although CTCs have been mainly isolated from carcinomas through epithelial antigen-targeted antibodies (EpCAM), reports have demonstrated that EpCAM expression is not restricted to epithelial-derived tumors, but shared as well by CTCs isolated from patients with mesenchymal-derived cancers [7]. In the present study, we sought to investigate the feasibility to isolate CTCs from a patient with secondary liver hemangiopericytoma using CellSearch system, the only FDA-approved for CTCs enumeration. CellSearch captures cells using a monoclonal antibody specific to EpCAM and identifies CTCs using differential fluorescent antibodies to detect the presence of cytokeratins (CK) within a nucleus-containing intact cell and absence of CD45, as defining characteristics of CTCs (DAPI+/CK+/EpCAM+/CD-45-).

## 2. Materials and Methods

### 2.1. Circulating Tumor Cells Detection

Circulating tumor cells were detected through two methods, one EpCAM-dependent (CellSearch) and the other size based (ScreenCell). Peripheral blood samples were drawn after informed consent into two tubes, after discarding the first milliliters to avoid contamination with cutaneous epithelial cells taken by the needle during the sampling. 7.5 mL of blood was collected in CellSave preservative tube (Menarini Silicon Biosystems, Castel Maggiore, Bo, Italy) and processed by CellSearch (Menarini Silicon Biosystems), employing CellSearch CTC kit, according to manufacturer's instructions. Six milliliters of blood was collected in tube containing K_2_EDTA and processed by ScreenCell Cyto kit (ScreenCell, Sarcelles, France) to isolate fixed cells for cytological studies. Briefly, in order to fix the cells and lyse red blood cells, 3 mL of blood was diluted in 4 mL of FC2 filtration buffer (ScreenCell). After 8 min of incubation at room temperature, 7 mL of diluted sample was added into device tank and filtered under a pressure gradient using a vacutainer tube. After washing with PBS to remove red blood cells debris, the filter was left on absorbing paper to dry at room temperature and then stored at −20°C. The filtration was carried out in duplicate and completed within 3 min.

### 2.2. Immunofluorescence Analysis on CTCs

For immunofluorescence, nonspecific protein binding was blocked by 5% normal goat serum. Specimens were permeabilized in PBS-Tween 20 (PBS-T) for 20 min.

Protocol 1: the filter was first incubated overnight at 4°C with primary mouse monoclonal anti-EpCAM (VU1D9; 1 : 50; Invitrogen Ltd., Paisley, UK) antibody and then, after washing twice with PBS-T, probed for 1 h with antimouse AlexaFluor-488 secondary antibody (1 : 50, Invitrogen). Next, section was incubated overnight at 4°C with staining solution of phycoerythrin- (PE-) conjugated cytokeratins (CK) 8, 18, and 19 and allophycocyanin- (APC-) conjugated CD45 antibodies (CellSearch CTC kit). Nuclei were counterstained with 4′,6-diamidino-2′-phenylindole (DAPI).

Protocol 2: the filter was first incubated overnight at 4°C with primary mouse monoclonal anti-EpCAM and rabbit polyclonal anti-CD45 (1 : 50; Cell Signaling Technology, Danvers, MA, USA) antibodies and then, after washing twice with PBS-T, probed for 1 h with antimouse AlexaFluor-350 and antirabbit AlexaFluor-594 (1 : 50; Invitrogen) secondary antibodies. Next, section was incubated overnight at 4°C with CD34 class III/FITC (Dako North America Inc., Carpinteria, CA, USA). Finally, specimen was counterstained with DRAQ5 (Cell Signaling Technology), a cell permeable far-red fluorescent DNA dye, for visualization of cell nuclei.

All primary antibodies were diluted in 1% bovine serum albumin in PBS-T.

The filters were scanned by a digital scanner (Aperio ScanScope FL System, Aperio Technologies, Inc., Oxford, UK) and were also analyzed by confocal microscopy (Leica TCS-SP2).

CTC clusters were defined as groups of CTCs containing ≥3 distinct nuclei according to previous publication [8]. In representative images, different pseudocolors were arbitrarily assigned, irrespectively to the actual fluorophore, to each of the four fluorescence channels and changed to optimize the visualization of the different signals. When displayed in the image, the nuclei were always showed in blue.

## 3. Results and Discussion

**Figure 1:**
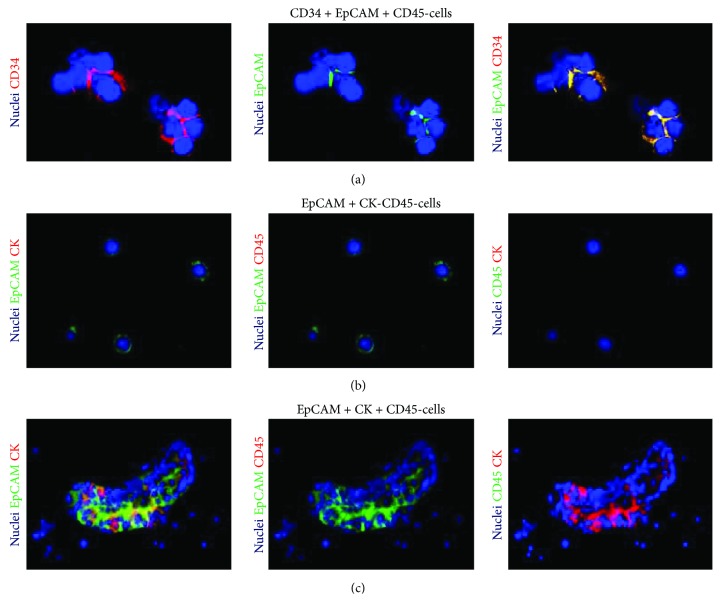
Phenotype of circulating tumor cells isolated using ScreenCell Cyto kit. In representative images, nuclei are displayed in blue. Pseudocolors were changed for each of the four channels to optimize the visualization of the different signals. (a) Immunofluorescence for CD34, EpCAM, and CD45. Clusters of CD45- cells coexpressing EpCAM and CD34 are displayed. (b) and (c) Immunofluorescence for EpCAM, cytokeratins (CK) 8, 18, and 19, and CD45. Single CD45-/CK- cells expressing EpCAM (b). Cluster of CD45- cells coexpressing EpCAM and CK (c).

According to the mesenchymal nature of hemangiopericytoma, we failed to detect typical epithelial-like CTCs, but we were able to identify 5 DAPI+/EpCAM+/CK-/CD45- events, which might be referable to cells with nonepithelial features. According to the CellSearch guidelines, all objects with no clear cytokeratin staining are defined as “suspicious objects.” Similar events have been previously described by our group to have prognostic significance in other cancer types, such as renal cell carcinoma, frequently lacking cytokeratin expression [9]. In order to better characterize these atypical CTCs, we processed a second blood sample by using ScreenCell, a device that allows a size-selective separation of CTCs from other blood cells. To evaluate the molecular profile of atypical CTCs, an immunofluorescence staining was performed on two filters obtained from the blood of the patient, using in the first one the same antibodies used in the CellSearch system (EpCAM, CK, and CD-45, as defining characteristics of CTCs) and in the second one CD34 (as hemangiopericytoma specific marker) (Figure 1) [10, 11]. In both the examined filters, single or clustered CTCs were identified and an average of one cluster per filter was observed. CTCs were variably round or elongated, presented a high nucleus to cytoplasm ratio, scant cytoplasm, and an oval nuclei. In examined filters, both single and clustered CTCs were found positive for EpCAM but negative for CD45. EpCAM expression was localized at the cell surface in single cells, but retained in the internal part of the clusters. The absence of EpCAM at the cluster surface might explain the failure of CellSearch to detect CTC clusters, as already demonstrated in epithelial-like tumors, such as nonsmall cell lung cancer [12]. EpCAM-positive clusters variably expressed CK but were constantly negative for CD45 and positive for CD34. Some studies have demonstrated that CTC clusters are composed of a number of tumor cells mixed to nontumor cells such as mesenchymal cells, epithelial cells, pericytes, immune cells, platelets, and cancer-associated fibroblasts [13]. In our patient, the coexpression of EpCAM, CK, and CD34 within the CTC cluster confirms the great heterogeneity in CTC clusters and their patent multifaceted composition. The feasibility of a liquid biopsy in a rare tumor, such as HPC, might have several crucial clinical implications. First, most intracranial meningeal HPCs resemble meningiomas in their clinical presentation and histological features and may therefore be misdiagnosed, despite important differences in prognosis [14]. Keeping scanning and following up with a noninvasive tool, such as a blood draw, might make the difference. Second, systemic therapy options for HPC are limited so far, and due to the increasing number of targeted therapies available, the molecular characterization of CTCs has the unique potential to translate into personalized treatments [15]. To this purpose, clinical trials on rare cancers are not usually performed due to the small numbers of eligible patients, and while more common cancers are increasingly being treated according to a biomarker-driven approach, the “one size fits all” approach is still the standard of care for rare tumors. Personalized targeted treatments need to be developed specifically for hemangiopericytoma as well as in all other rare cancers, and liquid biopsy has certainly the right credentials to facilitate this process.
